# Dynamic Behavior of *Salmonella*-Induced Membrane Tubules in Epithelial Cells

**DOI:** 10.1111/j.1600-0854.2008.00830.x

**Published:** 2008-10-20

**Authors:** Dan Drecktrah, Seamus Levine-Wilkinson, Tapen Dam, Seth Winfree, Leigh A Knodler, Trina A Schroer, Olivia Steele-Mortimer

**Affiliations:** 1 Laboratory of Intracellular Parasites, NIAID, National Institutes of Health, Rocky Mountain LaboratoriesHamilton, MT 59840, USA; 3 Department of Biology, The Johns Hopkins University3400 North Charles Street, Baltimore, MD 21218, USA

**Keywords:** confocal, endosomes, lysosomes, microtubule, *Salmonella*-containing vacuole, Sifs

## Abstract

*Salmonella* Typhimurium is a facultative intracellular pathogen that causes acute gastroenteritis in man. Intracellular *Salmonella* survive and replicate within a modified phagosome known as the *Salmonella*-containing vacuole (SCV). The onset of intracellular replication is accompanied by the appearance of membrane tubules, called *Salmonella*-induced filaments (Sifs), extending from the SCV. Sifs are enriched in late endosomal/lysosomal membrane proteins such as lysosome-associated membrane protein 1, but their formation and ability to interact with endosomal compartments are not characterized. In this study, we use live cell imaging techniques to define the dynamics of Sif formation in infected epithelial cells. At early time-points, Sifs are simple tubules extending from the surface of SCVs. These tubules are highly dynamic and exhibit bidirectional, microtubule-dependent movement. At the distal ends of individual Sif tubules, furthest from the SCV, a distinct ‘leader’ domain was often observed. At later times, Sifs develop into highly complex tubular networks that extend throughout the cell and appear less dynamic than nascent Sifs; however, individual tubules continue to display bidirectional dynamics. Sifs can acquire endocytic content by fusion, indicating a sustained interaction with the endocytic pathway. Together, these results show that these *Salmonella*-induced tubules form a highly dynamic network that involves both microtubule-dependent motility and interactions with endosomal compartments.

*Salmonella enterica* is a Gram-negative enteric pathogen that is closely related to *Escherichia* spp., *Shigella* spp. and *Citrobacter* spp. There are more than 2000 *S. enterica* serovars, many of which can cause disease in man and other animals. *S. enterica* serovar Typhimurium is one of several ubiquitous, nonhost adapted serovars that commonly cause acute self-resolving gastroenteritis in healthy adult humans. Like other pathogenic *Salmonella*, serovar Typhimurium is a facultative intracellular pathogen whose ability to survive within eukaryotic host cells is a crucial virulence determinant. Intracellular *Salmonella* reside in a modified phagosome known as the *Salmonella*-containing vacuole or SCV. Maturation of the SCV is characterized by the transient presence of early endosomal membrane proteins, which are rapidly replaced by membrane proteins normally found on late endosomes/lysosomes (LE/Lys) (1,2). The process of SCV maturation also involves a spatial shift from the site of internalization at the plasma membrane to the juxtanuclear region adjacent to the microtubule-organizing center (MTOC) (3). These initial maturation steps involve direct interactions with endocytic compartments and are similar to canonical phagosome biogenesis. The feature that most clearly distinguishes the SCV from other bacterial vacuoles or phagosomes is an extensive network of membrane tubules known as Sifs, whose appearance coincides with the onset of bacterial replication several hours after invasion (4). Sif tubules extend from the surface of the SCV and appear to be derived from late endocytic compartments; they contain the lysosome-associated membrane proteins (LAMPs), vacuolar adenosine triphosphatase (vATPase) and lysobisphosphatidic acid as well as cathepsin D (4–6).

The interactions between *Salmonella* and host cells are mediated largely by two specialized type III secretion systems, T3SS1 and T3SS2, which enable the bacteria to deliver distinct sets of effector proteins directly into the host cell. T3SS1 translocates effectors primarily across the plasma membrane and is essential for invasion of nonphagocytic cells. T3SS2, by contrast, operates intracellularly to translocate effectors across the SCV membrane. Several T3SS2 effectors, plus a variety of other *Salmonella* proteins, have been implicated in Sif formation, and a growing body of evidence suggests that they act by altering the host cell molecular machinery that drives the movement of endosomes and lysosomes along microtubules. Localization of the SCV at the MTOC, a process that precedes Sif formation, requires two T3SS2 effectors, SseF and SseG (7). Subsequently, Sif tubule formation involves the activities of at least two more effector proteins, SifA and PipB2. *Salmonella* mutants lacking SifA are unable to make Sifs or maintain the integrity of the SCV membrane and consequently escape into the cytosol (6). In contrast, PipB2 mutants remain within the SCV and can form Sifs; however, the length of Sif tubules is reduced compared with those formed by wild-type *Salmonella*(8). Although the molecular mechanisms involved in these processes remain obscure, both effectors appear to target microtubule-based motors. SifA has been shown to stimulate Rab7 uncoupling from dynein/dynactin and to modulate kinesin-1 association with the SCV (9,10). PipB2 has been shown to interact directly with the light chain subunit of the kinesin-1 motor complex and by itself causes translocation and accumulation of LAMP1-positive endosomes and/or lysosomes to the cell periphery (8,11).

Live cell imaging is a powerful tool that can provide new and unexpected insight into many subcellular behaviors. Previously, we used such methodology to show that SCV biogenesis is a highly dynamic process that involves extended interactions with multiple components of the endocytic pathway (2). In the present study, we use live cell imaging to obtain a detailed picture of Sif tubule extension, dynamics and network formation. We find Sifs to be highly motile, microtubule-dependent structures that engage in extension, branching and retraction at speeds consistent with microtubule-based transport. We show that Sif tubules can associate and fuse with membranes of the endocytic pathway, suggesting that membrane is recruited by fusion with existing compartments. Our analysis also revealed that some Sif tubules contain a structural subdomain at the distal end, furthest from the SCV, that may be a specialized site for interactions with endosomes, microtubules and motors. This study provides the first description of the dynamic behavior of Sifs in living cells and demonstrates that network formation involves both microtubule-dependent motility and membrane fusion events.

## Results

**Figure 1:**
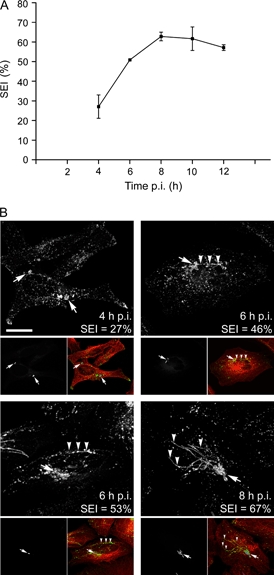
Sif network formation in *Salmonella*-infected HeLa cells HeLa cells grown on glass coverslips were infected with cherry-*Salmonella*, fixed at increasing times p.i. and then processed for analysis by scanning confocal microscopy (Zeiss LSM510). Antibodies to LAMP1 and β-tubulin were used to reveal SCVs/Sifs and microtubules, respectively. A) The SEI (mean ± SD) (8) is a quantitative measure of Sif tubule and network extension. The 20–30% SEI seen at 4 h p.i. represents a lower threshold. To have a SEI lower than this, a cell would have to contain either a single bacterium or a single tight cluster because the distance between individual SCVs contributes to the SEI. The 20–30% SEI values measured at 4 h p.i. illustrate the fact that most infected cells contained two to four bacteria in separate SCVs distributed throughout the cell. B) Representative images (projections of *z*-series) of *Salmonella*-infected cells at various times p.i. are provided to show the variation in Sif morphology. The large panels show LAMP1 staining and provide the SEI value for the cells shown and the time p.i. (scale bar = 20 μm). The smaller panels show the same cells and illustrate the distribution of cherry-*Salmonella* (greyscale images, left) plus all three markers (*Salmonella* in blue, LAMP1 in green and β-tubulin in red). Arrows indicate the location of intracellular *Salmonella* in SCVs, and arrowheads indicate Sif tubules.

Immunofluorescence studies of fixed *Salmonella*-infected cells have shown that LAMP-enriched Sifs can be detected at 6 h post-infection (p.i.) or later (4,8,10,12). To precisely delineate the kinetics of Sif formation, we determined the extent of Sif formation at different time-points following infection. HeLa cells were infected with *S. enterica* serovar Typhimurium SL1344 expressing the red fluorescent protein mCherry (cherry-*Salmonella*) for 10 min (13). Cells were then fixed at 4, 6, 8, 10 or 12 h p.i. and stained with antibodies to LAMP1 and β-tubulin to reveal SCVs/Sifs and microtubules, respectively. Tubulin staining provided an indicator of microtubule integrity and also helped to define the overall contours of each infected cell. Cells were imaged on a confocal microscope, and Sif formation was assessed using the Sif extension index (SEI), a measure of the area occupied by the SCV and Sifs (LAMP1 staining) expressed as a percentage of the area occupied by β-tubulin staining (8). Figure 1 shows that Sif networks were maximally extended by approximately 8 h p.i., by which time many Sifs are extensive. However, the aggregate length of individual Sif tubules is still heterogeneous at this time, and some cells contain either no Sifs or very simple Sifs. Because this is not apparent from Figure 1A, which shows the mean ± SD for three independent experiments, representative Sifs are also shown [Figure 1B; (12)]. These images also show that Sif morphology is heterogeneous; some cells contain clearly defined contiguous tubules, whereas others have discontinuous linear arrays of vesicles, also referred to as ‘beads-on-a-string’(14,15), interspersed with tubular profiles (arrowheads in Figure 1B).

**Figure 2:**
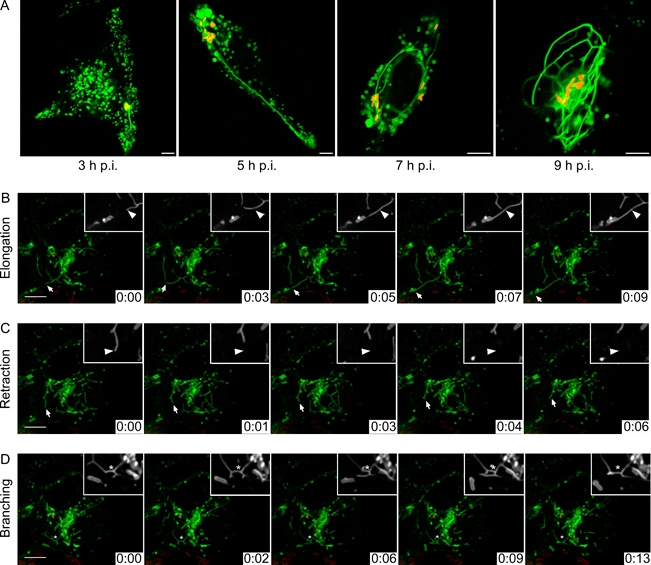
Live cell imaging of Sifs reveals a dynamic tubular network A) Time–course of Sif network formation in live HeLa cells. HeLa cells transiently expressing NPC1-eGFP were infected with mRFP-*Salmonella*. Infected HeLa cells were imaged at indicated times using a point-scanning confocal microscope. Single 1 μm confocal images are shown; eGFP-NPC1 (green) and *Salmonella* (red). Scale bar = 5 μm. B–D) HeLa cells transiently expressing LAMP1-mGFP were infected with cherry-*Salmonella* for 6 h and then imaged using a spinning disk confocal microscope. See also Movie S1. Processed images from a time-lapse series from one single cell are shown. The larger panels show *Salmonella* (red) and LAMP1-mGFP (green). Scale bar = 10 μm. The elapsed time (min:seconds) is indicated in the lower right hand corner of each panel. The small panels show LAMP1-mGFP in greyscale. B) A Sif tubule (arrowhead) elongates. C) A Sif tubule (arrowhead) retracts. D) Branching dynamics in Sif tubules (asterisk).

Conventional immunofluorescence localization of proteins in fixed cells is known to be a poor reporter of tubular membrane compartments, and the fragmented appearance of some Sifs suggested to us that not all tubules are properly preserved during fixation (15). If true, this could confound Sif identification, particularly at early time-points when tubules tend to be short. To address this concern and learn more about the dynamic events that underlie Sif network formation, we analyzed Sifs in living, *Salmonella*-infected HeLa cells transiently expressing the LE/Lys markers Nieman Pick Type C protein (NPC1)-enhanced green fluorescent protein (eGFP) or LAMP1-monomeric GFP (mGFP) (Figure 2). Both markers have been shown to be faithful reporters of LE/Lys dynamics that have no discernible effect on compartment behavior (16–18). In our previous study of SCV biogenesis, we used a Yokogawa spinning disk confocal optimized for speed and sensitivity (2). For the present analysis, we used this system in conjunction with point-scanning confocal and wide field fluorescence microscopy.

In HeLa cells infected with *Salmonella* [cherry-*Salmonella* or monomeric red fluorescent protein (mGFP)-*Salmonella*], live cell imaging revealed some Sif tubules as early as 3 h p.i. (Figure 2A shows mRFP-*Salmonella*). At this stage, Sifs were typically short, simple tubular structures that rarely extended more than 5 μm from the SCV. Although structurally simple, the tubules formed during 3–6 h p.i. were highly dynamic and most underwent multiple episodes of extension, retraction and pausing (Figure 2B and Movie S1). At later times, Sifs formed complex, extensively branched networks that extended throughout the cell and in many cells appeared to wrap around the nucleus (Figure 2A). The trend toward increasing complexity continued until approximately 9 h p.i., by which time the Sifs in many cells had formed an extensive, interconnected network of tubules. In the course of our analysis, we also noted that the extent and complexity of the Sif network correlated with a decrease in the number of free LAMP1-mGFP and NPC1-eGFP vesicles. By 7–9 h p.i., very few free vesicles were observed and both markers were now predominantly localized to dynamic tubular structures (Figure 2A, compare 3 with 7 or 9 h time-points).

**Figure 3:**
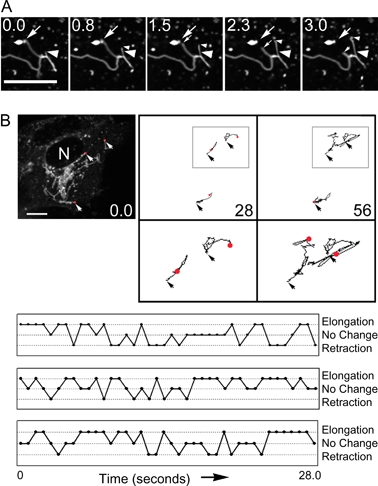
Sif tubule extension involves bidirectional movement HeLa cells expressing LAMP1-mGFP were infected with cherry-*Salmonella* for 6 h and then imaged using a spinning disk confocal microscope. A) A series of single optical planes taken from a time-lapse series showing Sifs that undergo both elongation (large arrowhead indicates starting position and small arrowhead follows the end of the tubule) and retraction (large arrow indicates starting position and small arrow follows the end of the tubule). Scale bar = 5 μm. B) Sif extension is bidirectional and involves switching between elongation and retraction. One representative image (single confocal section) from the beginning of an 80 image time series (0.7 seconds apart) is shown. N, nucleus; scale bar = 5 μm. The distal ends of three Sif tubules were then tracked (ImageJ macro Manual Tracking) over the subsequent frames, and the tracks at 28 and 56 seconds are shown in the panels on the right. For each track, an arrow indicates the initial position of the tubule tip and a red dot indicates the final position. Enlargements of the tracks bound by the grey box are shown in the lower panels. The bottom panel provides graphic representations of the behavior of three separate Sif tubules that illustrates switching between extension, retraction and pauses.

Analysis of time-lapse image sequences, acquired at one image per 0.7 seconds, revealed that Sif extension is an iterative, intermittent process characterized by bidirectional movement of individual tubules (Figure 3B). Tubule ends switch frequently between periods of extension, retraction and oscillation, and they can change direction as seen when the tubule end is tracked (Figure 3B). Tubule extension and retraction can occur simultaneously at the ends of branched tubules emanating from a single ‘parental’ tubule (Figure 3A). Importantly, all Sifs seen in living cells were continuous tubules and the beads-on-a-string appearance seen in fixed cells (4,5) was never observed, strongly suggesting that this phenotype is an artifact of fixation.

The dramatic changes in Sif network architecture over time prompted us to explore the dynamics of individual Sif tubules in detail. For optimal sensitivity and speed, we utilized high-speed wide field microscopy to measure the extension and retraction velocities of individual Sifs. The tips of rapidly extending Sif tubules, which exhibited steady, relatively uniform movement, moved at a mean velocity of 0.44 ± 0.14 μm/second (Table 1). In contrast, tubule retraction appeared to occur through two distinct mechanisms. Some tubules retracted steadily over a period of several seconds (mean velocity 0.68 ± 0.25 μm/second), whereas others retracted very rapidly over a short time frame (2.13 ± 0.56 μm/second) in a manner suggestive of elastic recoil.

**Table 1:** Parameters of Sif dynamics^a^

		*n*	Velocity (μm/second)			t-test p value
			Mean	Range	SEM	
Extension	Lifespan 0–600 seconds	33	0.44	0.27–0.84	0.02	
	Lifespan >600 seconds	24	0.33	0.21–0.45	0.01	0.0001
Retraction	Slow	54	0.68	0.25–1.53	0.03	
	Rapid	17	2.13	1.5–3.01	0.13	2.98E-06

a HeLa cells transiently expressing NPC1-eGFP were infected with a pulse of mRFP-*Salmonella* and then imaged at 4–8 h p.i. using a wide field microscope (3i Marianas) at one frame/second for 15–25 min. Sif extension and retraction velocities were quantified using the ImageJ macro Manual Tracking.

**Figure 4:**
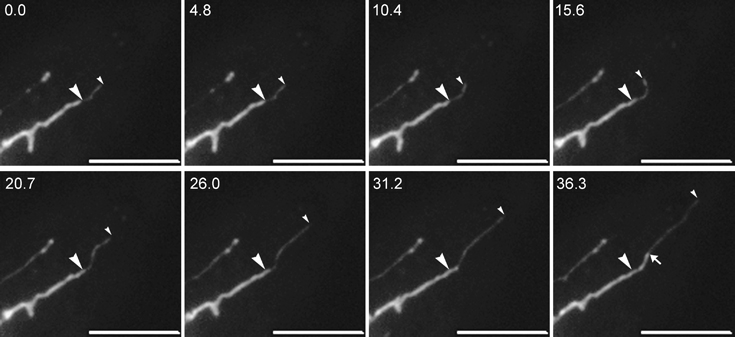
Appearance and extension of a leader Sif HeLa cells expressing NPC1-eGFP were infected with mRFP-*Salmonella* for 6 h and then imaged using a wide field microscope. Processed time-lapse images from a single cell are shown. The elapsed time (in seconds) is indicated at top of each image. The top four images (0–15.6 seconds) show a leader Sif (small arrowhead) extending from a stationary trailing Sif (large arrowhead). The bottom four images (20.7–36.3 seconds) show the trailing Sif (large arrowhead) extending into the region previously occupied by the leader Sif. The arrow in the last panel marks the junction between the leading and the trailing Sif. Scale bar = 10 μm.

Closer inspection of Sifs in cells expressing either LAMP1-mGFP or NPC1-eGFP revealed the presence of a distinct subdomain at the distal end (furthest from the SCV) of some Sif tubules. This domain appeared considerably less bright than the remainder of the tubule (Figure 4), suggesting that the amounts of these two LE/Lys membrane marker proteins are decreased compared with the main body of the tubules. These ‘leader’ Sifs, which were observed routinely, are transient structures that persist for up to 5 min before retracting back into or being overtaken by the trailing Sif. Multiple rounds of leader Sif growth and retraction could be observed from a single trailing Sif. The boundary between a leader Sif and its trailing domain did not appear to be fixed, and each domain was capable of extending independently, or in tandem with, the other (Figure 4, last two images in the time series).

**Figure 5:**
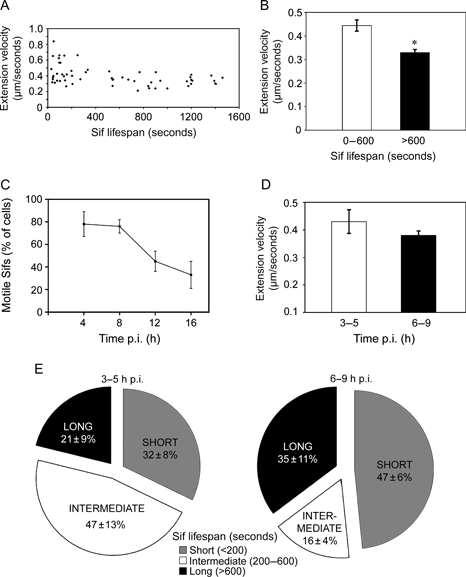
Analysis of Sif extension velocity and lifespan as a function of time p.i HeLa cells transiently expressing NPC1-eGFP were infected with mRFP-*Salmonella* and imaged using a wide field microscope (3i Marianas) at one frame/second for 15–25 min. Sif extension and retraction velocities were quantified using the ImageJ macro Manual Tracking. A) Mean extension velocities plotted against the lifespans of individual Sifs. B) Comparison of the mean (±SEM) extension velocity for all Sifs with 0–600 second life spans (white bar) and >600 second life spans (black bar). Asterisk indicates that mean extension velocities are significantly different. C) Sif dynamics in the cell population as a function of time p.i. Cells that contained discernable Sifs were observed for 1 min each. A cell was scored positive for motile Sifs if any Sif movement was observed within this time frame. The percent of the cell population showing motile Sifs is shown. D) Mean (±SEM) extension velocities of Sifs observed at 3–5 h p.i. (white bar) and 6–9 h p.i. (black bar). The values are not significantly different. E) Relative proportions of Sifs with lifespans <200 seconds (short; grey), 200–600 seconds (intermediate; white) and >600 seconds (long; black) at 3–5 and 6–9 h p.i. The mean ± SD from four independent trials is shown; *n*≥ 6 Sifs were evaluated for each condition.

During the course of our imaging analysis, we noted that some Sif tubules persisted for brief periods of time, whereas others appeared very long lived. To investigate this phenomenon further, we first asked whether the lifespan of a Sif might correlate with its extension velocity, a link that would not have been apparent in our initial characterization. Indeed, when these two parameters were plotted against each other, a clear relationship emerged (Figure 5A). Tubules with a lifespan of ≥10 min extended at a lower mean rate (0.33 ± 0.02 μm/second) than tubules with shorter lifespans (0.44 ± 0.14 μm/second; Figure 5B and Table 1). In other words, shorter lived tubules extended faster than longer lived tubules.

At early times p.i., Sifs appeared to be consistently shorter and more dynamic (see above), whereas longer, more stable tubules appeared to become predominant later in infection. In some cells, the complex tubular networks seen at late times p.i. (6 h and beyond) appeared quiescent, suggesting that tubule motility had ceased altogether. To determine whether this was indeed the case, we evaluated the motility of Sif networks in living cells over time. Sif extension [as determined by making SEI estimates on live cells viewed by differential interference contrast (DIC) microscopy] increased over the course of infection (data not shown), as expected from our analysis of fixed cells (Figure 1). The percent of cells that showed Sif tubule dynamics decreased gradually over this same time period, but considerable motility was still observed even as late as 14 h p.i. (Figure 5C).

The accumulation of slow or nonmoving Sifs and tubular Sif networks at later times p.i. might reflect a global change in cellular physiology, such as downregulation of microtubule-based motor activity that could be a direct consequence of *Salmonella* infection. Under these circumstances, Sif lifespans and extension velocities would be predicted to change as a function of time p.i. To explore this possibility, we evaluated the mean extension velocity and lifespan of Sifs in cells at early (3–5 h) versus later (6–9 h) p.i. These intervals were chosen because they represent times when Sifs are obviously actively extending (3–5 h p.i.) and times when Sif extension has reached a maximum (6–9 h p.i.). When we compared Sif tubule dynamics at these two times, we detected no significant difference in either mean extension velocity (Figure 5D and Table 1) or lifespan (data not shown), indicating that the properties of individual moving Sifs does not change as a function of time p.i.

These data indicate that Sif tubules remain motile at late times p.i., yet their overall organization changes dramatically (Figure 2A). In trying to resolve this issue, we reasoned that the development of extensive, interconnected Sif networks might be the result of a switch from predominantly short-lived, rapidly extending tubules to a longer lived, slowly extending population. To determine whether this was indeed the case, we categorized Sifs into three groups according to lifespan: short lived (<200 seconds), intermediate (200–600 seconds) and long lived (>600 seconds) and then compared the proportions of the three groups at early (3–5 h) and late (6–9 h) times p.i. We found that the proportion of long-lived Sifs did increase as a function of time p.i.; during the early interval, ≈20% of Sifs were long lived, whereas ≈35% were long lived at the later times (Figure 5D). However, the population of short-lived Sifs was also more predominant at later times (≈50 versus ≈30%). Thus, the proportion of both long-lived and short-lived Sifs increased at the expense of Sifs with an intermediate lifespan. Taken together, our findings suggest that the establishment of Sif networks involves an increase in stability of some tubules but not a complete suppression of Sif extension.

We extended this work by evaluating the overall motility of Sif networks over time (Figure 5). For this experiment, we examined Sif extension and movement in living cells. Sif extension (as determined using the SEI) increased over the course of infection (not shown), as expected from our analysis of fixed cells (Figure 1). The percent of cells that showed Sif tubule dynamics decreased gradually over this same time period, but considerable motility was still observed even as late as 16 h p.i. (Figure 5C).

**Figure 6:**
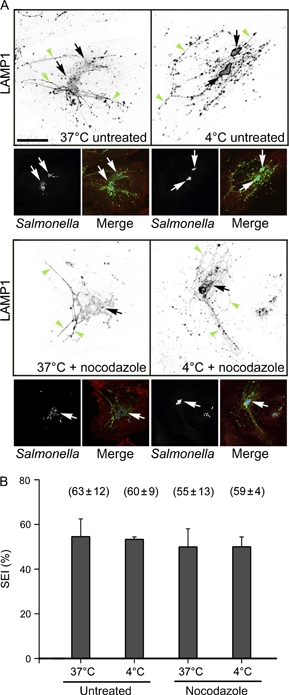
Sifs persist in the absence of microtubules HeLa cells were infected with cherry*-Salmonella* and incubated for 8 h to allow for Sif formation. 37°C untreated: cells were then fixed in paraformaldehyde (PFA) for 10 min at 37°C. 37°C + nocodazole: cells were treated with nocodazole (10 μg/mL) for 15 min and then fixed in PFA with nocodazole for 10 min at 37°C. 4°C untreated: cells were shifted to ice for 15 min and fixed in PFA on ice. 4°C + nocodazole: cells were shifted to ice and treated with nocodazole for 15 min and fixed in PFA with nocodazole on ice. Subsequently, cells were processed for immunofluorescence with antibodies to LAMP1 and β-tubulin. A) Shown are *z*-projections of representative cells. Sif tubules were stained with anti-LAMP1 antibody (large panels), and the greyscale images have been inverted to allow for better resolution of tubules and vesicular structures. The small panels show the location of cherry-*Salmonella*(left and merge) and microtubules (merge). Arrowheads indicate LAMP1-positive tubules or linear arrays of LAMP1-positive vesicles. Arrows indicate the location of intracellular *Salmonella*. B) Quantification of Sif extension in treated cells. SEI was determined from 20 cells per treatment. Error bars represent the SD of three independent experiments. No significant difference was detected (one-way ANOVA with Dunnett's *post hoc*test). The mean ± SD percentage of infected cells containing Sifs for each treatment is shown in parentheses.

Pretreatment of cells with the microtubule-depolymerizing agent, nocodazole, prevents Sif formation (4,14), presumably because tubule extension occurs along microtubules. However, the question of how microtubules contribute to the motility of preformed Sif tubules has never been addressed. To investigate this, we allowed Sif networks to form in infected HeLa cells for 8 h p.i. and then incubated the cells for 15 min in nocodazole (10 μg/mL) to disrupt microtubules. Live cell imaging confirmed that this treatment caused a complete cessation of movement of endocytic vesicles containing internalized fluorescent dextran, confirming that microtubules were depolymerized, and also had a dramatic effect on the dynamics of Sif tubules (Movie S2 and data not shown). Elongation and retraction ceased completely, and the Sif tubules appeared essentially ‘frozen’ in place even though microtubules were largely disassembled (Figure 6). This effect is reversible because tubule dynamics were rapidly restored following washout of nocodazole (data not shown). To confirm this result, we also used exposure to low temperature to depolymerize microtubules. In cells incubated on ice (15 min) and/or treated with nocodazole, we observed no change in either the SEI or the percentage of infected cells that contained Sif networks (Figure 6B). This result suggests that the Sif network could be stabilized by factors other than microtubules. An obvious candidate for this role is actin because, although it is not required for Sif formation (19), actin remodeling is required for intracellular replication of *Salmonella* in epithelial cells (20). To test this possibility, we treated cells concurrently with nocodazole and Latrunculin A (1μM), an agent that favors actin disassembly (21). Sifs persisted after this treatment as well (data not shown), indicating that their stability depends on neither actin nor microtubules.

**Figure 7:**
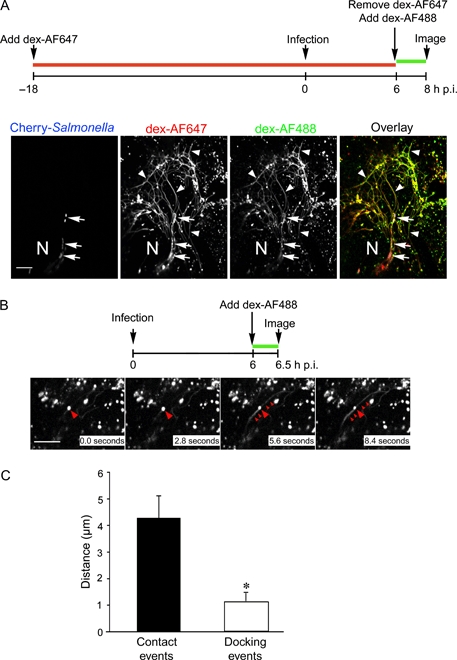
Imaging of Sifs loaded with endocytosed dextrans A) HeLa cells were loaded with fluorescent dextrans and infected with cherry-*Salmonella* as described in the text (for clarity, a graphic depicting the experimental scheme is shown). Cells were then imaged using a spinning disk confocal microscope. A single processed image from a time-lapse series is shown. N, nucleus. Arrowheads indicate tubules containing both fluorescent dextrans. Arrows indicate the location of intracellular *Salmonella*. B) To visualize delivery of incoming endocytic content to Sifs, cells were infected with *Salmonella* and dex-AF488 was added as a 30-min pulse at 6 h p.i. as depicted in the graphic showing the experimental scheme. A series of images is shown; elapsed time is indicated. Between 2.8 and 5.6 seconds, a dex-AF488-loaded vesicle (large arrowhead) can be seen delivering its content to a pre-existing Sif tubule (indicated with small arrowheads) (see also Movie S3). Scale bar = 10 μm. C) Quantification of the transient (contact; black bar) versus long-term (>20 seconds; docking; white bar) interactions of endosomes with Sif tubules as a function of distance from the end of the tubule. Values are mean ± SEM. Because leader Sifs are much dimmer than vesicles, they could not be readily seen in this analysis, so the distance measurements are assumed to be relative to the end of the trailing Sif domain. Asterisk indicates that the mean distances are significantly different.

Sifs emanate from a parent compartment, the SCV, which is accessible to incoming endocytic markers and can fuse directly with lysosomes containing fluorescently labeled dextrans as soluble content markers (2). In this study, we used a similar approach to investigate whether Sif tubules are similarly able to fuse with components of the endocytic pathway. Cells were incubated overnight with Alexa Fluor 647-conjugated dextran (dex-AF647), then infected with cherry-*Salmonella* and incubated for an additional 6 h in the presence of dex-AF647. Dex-AF647 was then removed, and cells were incubated for two more hours in the presence of a second color dextran, dex-AF488. Live cell imaging was initiated as soon as the excess dextran was removed by washing. Under these conditions, almost all the Sif tubules we observed contained both fluorescent dextrans (Figure 7), indicating that Sif biogenesis involves significant interactions with endocytic organelles. Because dex-AF647 was present continuously during the entire initial period of Sif formation, it may have been delivered to Sifs or the SCV from any element of the endocytic pathway. However, that dex-AF488 that had been taken up by cells starting at 6 h p.i. was also able to gain access to Sifs suggests that early and/or late endosomes might be able to deliver content to tubules by fusing with them directly. To test this hypothesis, we internalized dex-AF488 for a brief (30 min) pulse after Sifs were allowed to form and then looked for fusion events by live cell imaging. As shown in Figure 7B and Figure S3, dex-AF488-loaded endosomes were observed fusing with pre-existing Sif tubules and delivering their contents into the tubule lumen. These experiments demonstrate unequivocally that Sif tubules are accessible to, and in direct communication with, incoming endocytic traffic.

The observation that Sifs have two distinct domains, a leader Sif that contains a low density of NPC and LAMP1 and a trailing domain that is relatively enriched in these same proteins, raises the possibility that these membrane domains might show different interactions with free LE/Lys. We explored this possibility by examining interactions between Sifs and endosomes, which were labeled by internalization of fluorescent dextrans. The observed interactions were then categorized into two groups based on the duration of the interaction (Figure 7C). A ‘contact-only’ interaction was defined as an event in which an endosome contacted a Sif tubule transiently; in other words, an event where an endosome stopped moving when it encountered a tubule. ‘Docking’ interactions were defined as events in which endosomes underwent long-term (>20 seconds) interactions with a Sif tubule. We then determined where these two types of interactions occurred on the Sif. Long-term docking events were found to be more likely to occur near the distal end of Sifs (mean = 1.2 μm; *n* = 9 of 29 total contacts) compared with transient, contact-only events, which occurred farther from the tip (mean = 4.3 μm; *n* = 20 of 29 total contacts). Unfortunately, because fusion events were considerably harder to capture than either docking or contact-only events, we were unable to determine if these sustained interactions lead to later fusion events or if fusion is more likely to occur at certain regions of Sif tubules.

## Discussion

The induction of extensive tubular compartments in *Salmonella*-infected epithelial cells was first described 15 years ago (4,19). Originally identified as LAMP-enriched filaments that did not appear to be accessible to internalized endocytic markers, Sifs have now been clearly defined as tubular extensions of the SCV. Sif formation is intimately linked to the initiation of intracellular replication of *Salmonella*, yet surprisingly little is known about the mechanisms by which Sifs form. Their physiological function remains undetermined, although mutant *Salmonella* strains that cannot make Sifs have decreased virulence in mouse models of *Salmonella* infection, suggesting that the molecular changes that underlie Sif formation are necessary for pathogenesis (6,22). Sif formation appears to correlate with enhanced integrity of the vacuolar membrane because SifA mutants are seen to escape into the cytosol where, in macrophages, they are rapidly killed (23). An understanding of the molecular mechanisms that underlie Sif formation and how host cell and *Salmonella* effector proteins contribute is a critical foundation for future work on *Salmonella* pathogenesis.

In this study, we have investigated the formation of Sifs in *Salmonella*-infected epithelial cells using live cell microscopy. Our experiments reveal a dynamic process characterized by rapid, bidirectional tubule movement and branching that is accompanied by sustained interactions with the endocytic pathway. In addition, we have identified a novel subdomain at the distal end of Sif tubules that may contribute to motility and/or fusion.

Before Sifs are observed, the process of SCV maturation appears similar to canonical phagosome biogenesis, that is it involves the sequential acquisition of endosomal markers and leads to enrichment within 2 h in late endosomal/lysosomal membrane such as LAMP1 (1). In contrast, the formation of Sifs – a network of dynamic tubules extending from the surface of the SCV – is a visually dramatic departure from classic phagolysosome maturation. Previous work using fixed cells suggested that Sif formation did not begin until 6 h p.i., but our work reveals that Sifs form as early as 3 h p.i. This time frame suggests that Sif formation is concomitant with T3SS2 assembly (24). At these early times, the majority of Sifs are short lived and can be seen to extend, retract and make frequent stops and changes in direction. Sif formation requires the presence of an intact microtubule network (4,^14^,^19^,25), and the bidirectional nature of tubule movement suggests the involvement of distinct minus-end-directed and plus-end-directed motors. The velocity of extension of these short-lived tubules is in the range of 0.27–0.84 μm/second, with a mean velocity of 0.44 μm/second, similar to the speeds observed for late endosome and lysosomes (18,^26^–28). Candidate plus-end-directed motors include kinesin-1 and kinesin-2, both of which have been shown to be important for LE/Lys dynamics. Kinesin-1 is required for extension of tubular lysosomes (29–32), and kinesin-2, the major motor for the outward movement of pigment granules (33), was recently found to contribute to LE/Lys localization in nonpigmented cells (34). Of these two motors, only kinesin-1 has been directly implicated in Sif formation (11). Sif retraction appears to occur by two distinct mechanisms: one that is continuous and may be motor driven (mean velocity: 0.68 μm/second, range 0.25–1.53 μm/second) and a second very rapid type (mean velocity 2.13 μm/second) that appears to represent the precipitate recoil of a tubule under considerable tension. If a minus-end-directed motor mediates the slower retraction, the obvious candidate is cytoplasmic dynein, a protein known to drive the movement of a wide variety of endocytic structures and phagosomes along microtubules (35–37). Dynein has already been implicated in movement of the maturing SCV toward the MTOC (38) and Sif formation (39).

At later times p.i. (>8 h), Sif organization was clearly different. Instead of simple, individual tubules, Sifs are now organized into complex, interconnected networks. At first glance, the tubules that comprised these networks appeared considerably less dynamic than at earlier times, and closer examination revealed a population of long-lived (>10 min) tubules that moved slowly, if at all. However, a sizeable population of short-lived, dynamic Sifs was also observed. The overall proportion of the long-lived tubules increases with time, most existing tubules are now completely incorporated into the interconnected network and fewer free tubule ends are seen. Together, these features lead to a measurable decrease in tubule motility. However, our data show clearly that short-lived, highly motile tubules persist in a significant population of cells. One possible mechanism for the establishment of long-lived, relatively nonmotile Sifs is that the tubules have become engaged in interactions with cytoskeletal components. One possibility is intermediate filaments because we find that Sifs remain extended (yet motionless) when both microtubules and actin are acutely disrupted. An alternative explanation is that *Salmonella* may be able to stabilize microtubules or actin filaments. However, our microscopy studies did not reveal the presence of stabilized actin or microtubules in association with Sif tubules under these conditions. Sifs appear unlike most other tubular endomembrane systems in uninfected mammalian cells such as the ER and tubular lysosomes as these are highly dynamic and can be extremely transient in nature. Although Sifs bear a vague resemblance to the tubular lysosomes seen in activated macrophages or J774.2 macrophage-like cells, tubular lysosomes are completely destabilized and vesiculate when microtubules are disassembled (40).

The ability of *Salmonella* to survive for long periods of time within host cells, a phenomenon that is key to its success as an intracellular pathogen, has been attributed to its ability to impede lysosome fusion with the SCV (41–43). However, recent work from our laboratory has shown that SCVs interact with endolysosomal compartments in a sustained and dynamic fashion in epithelial cells (2). We now show that Sif tubules are also able to interact and undergo fusion with endosomes, similar to what has previously been shown for tubular lysosomes and macropinosomes (44). Our analysis of the spatial distribution of endosome/Sif interactions indicates that long-term docking events are most prevalent near the distal ends of tubules, farthest from the SCV. However, fusion events with tubules are rare, so it has not yet been possible to determine whether leader Sifs have a greater propensity for fusion. Despite the low incidence of observed fusion events along Sif tubules, Sif networks become consistently labeled with fluorescent endocytic probes, suggesting that the SCV itself may be the major site for fusion with incoming endosomes.

The role of lysosome fusion in SCV and Sif biogenesis remains controversial; however, we and others have found that lysosomes fuse efficiently with the maturing SCV (2,45). The live cell studies described herein, utilizing two distinct LE/Lys markers, reveal a dramatic depletion of these endocytic vesicles in cells that contain extensive Sif networks. Similar reductions in the number of LAMP-positive vesicles have also been observed in fixed *Salmonella*-infected cells [unpublished data and (46)]. Furthermore, the T3SS2 effector SifA, when transiently expressed in mammalian cells, causes aggregation and tubulation of LAMP-positive vesicles in the absence of any other bacterial factors (46–48). Together, these observations suggest that coalescence of LE/Lys is a crucial step in Sif formation and that ultimately most, if not all, LE/Lys material is incorporated into Sif networks. The implications of this are considerable for the host cell as well as the pathogen. What effect does LE/Lys aggregation and tubulation have on the ability of the host cell to sustain normal membrane trafficking pathways? Conversely, how do *Salmonella* survive within a vacuole that is formed by fusion with lysosomes? Resolving these questions will provide essential information about how the host–pathogen balance is maintained and has the potential to lead to identification of novel targets for antimicrobial therapies.

Taken together, the findings described in this study reveal that *Salmonella* induce the formation of a complex, highly dynamic, tubular endosome network in infected epithelial cells. Although the function of Sifs remains to be elucidated, some possible roles could be maintenance of the intracellular niche, providing membrane for the expanding SCV, procurement of nutrients (by the endocytic pathway) or dilution of lysosomal enzymes (by fusion of multiple endolysosomal compartments). An understanding of the molecular mechanisms that underlie Sif formation, and the roles played by host cell and *Salmonella* effector proteins, will provide the foundation for future work on *Salmonella* pathogenesis and is the subject of our ongoing work.

## Materials and Methods

### Cell culture, bacterial strains and reagents

HeLa (human adenocarcinoma cervix epithelial, CCL-2) were obtained from the American Type Culture Collection (ATCC) and grown in a humidified 37°C, 5% CO_2_ tissue culture incubator. Cell culture reagents were from Mediatech unless otherwise stated. HeLa cells were maintained in Eagle's MEM containing 2 mM L-glutamine, 1 mM sodium pyruvate and 10% heat-inactivated FBS. Low passage number (<25 after receipt from ATCC) cells were seeded on glass coverslips (Fisher Scientific) or Delta-T glass bottom dishes (BiopTechs) and grown overnight before infection.

Wild-type *S. enterica* serovar Typhimurium SL1344 (49) and *Salmonella* serovar Typhimurium 12023 (50) strains were previously described. To construct pFPV-mCherry/2, the oligonucleotides mCherry-FXba (5′ TGC TCT AGA TTT AAG AAG GAG ATA TAC ATA TGG TGA GCA AGG GCG AGG AG 3′) and mCherry-RSph (5′ CAT GCA TGC TTA CTT GTA CAG CTC GTC CAT 3′) (engineered restriction sites are underlined) were used to amplify mCherry from pRSET-B mCherry (51). The resulting amplicon was digested with *Xba* I and *Sph* I and ligated into the corresponding sites of pFPV25.1 (52), thereby replacing the *gfp mut3*gene. *Salmonella* harboring pFPV-mCherry/2 constitutively express mCherry under the control of the *rpsM*promoter and are not compromised for invasion or replication in HeLa cells (unpublished data). *Salmonella*expressing mRFP from pBR-RFP.1 were prepared as described (53). Chemicals were from Sigma unless otherwise noted. Stock solutions of 16 μM nocodazole in dimethyl sulfoxide were stored at −20°C. Fluorescent dextrans were from Invitrogen.

### Bacterial growth and infection

To infect epithelial cells, *Salmonella*were grown under invasion inducing conditions (1). Briefly, overnight shaking 37°C cultures in Luria-Bertani Miller broth (LB) were diluted in fresh LB and grown shaking at 37°C to late log phase. *Salmonella*were collected by centrifugation at 6000 × ***g***for 2 min and resuspended in Hank's buffered saline solution (HBSS) and used immediately to infect cells (multiplicity of infection ∼20–150) for 10 min at 37°C. Extracellular bacteria were then removed by aspiration and monolayers washed twice in HBSS. Infected cells were incubated for 1 h in growth media (GM) + 50 μg/mL gentamicin, to kill extracellular bacteria, and then in GM + 10 μg/mL gentamicin for the remainder of the experiment.

### Transient transfection of HeLa cells

For expression of LAMP1-mGFP (17), the plasmid was transfected into HeLa cells grown on Delta-T glass bottom dishes (BiopTechs) using Fugene 6 (Roche) according to the manufacturers instructions. For expression of NPC1-eGFP, HeLa cells were grown on 10-cm tissue culture dishes until they reached ∼70–90% confluency. Cells were then harvested using 0.05% trypsin–ethylenediaminetetraacetic acid, followed by centrifugation at 5000 × ***g***for 5 min. Pellets were resuspended in 500 μL Opti-MEM (Gibco BRL) and mixed with 15 μg NPC1-eGFP (18), followed immediately by electroporation (BTX ECM 600 settings: 270 V, 1500 μF, 129 Ω) (BTX). After 10 min recovery, cells were seeded onto 3.5-cm glass bottom tissue culture dishes (Mattek) at a low density (3–5 × 10^4^ cells/dish) and incubated overnight before infecting.

### Live cell imaging

For the spinning disk confocal system, cells seeded in Delta-T glass bottom dishes were grown overnight and then incubated with fluorescent dextrans (133 μg/mL). Cells were imaged in CO_2_-independent media (Invitrogen) containing 10% FBS and maintained at 37°C using a collar heater to warm the objective and a heated dish system (BiopTechs). Individual dishes were utilized for no more than 70 min. Images were collected using a Yokogawa spinning disk head mounted on a Nikon Eclipse TE2000-S inverted microscope with a 60×, 1.4 numerical aperture (NA) oil immersion objective (Nikon). Illumination was provided by a two-laser fiber optic laser launcher (Prairie Technologies Inc.) with krypton/argon (488 nm beam) and argon (568 and 647 nm beams) lasers selected by an acousto-optic tunable filter. Fluorescent emission (filters 525 nm/50, 600 nm/45 and 700 nm/75) was detected using a Photometrics Cascade II:512 camera (Princeton Instruments). A 1.5× magnifier lens positioned between the emission filter and the camera increased the size of the acquired image. Image acquisition was controlled by Metamorph® (Molecular Dynamics). Images were collected every 0.7 or 0.9 seconds.

HeLa cells expressing NPC1-eGFP were infected with mRFP-*Salmonella*12023 and then incubated at 37°C for 3 h before imaging. To assess the extent of Sif network formation in live cells, 1 μm optical sections were collected using point-scanning confocal microscopy (Zeiss; LSM 510; 63×, 1.4 NA oil immersion objective).

Sif tubule movement, lifespan and leader Sif measurements were collected using the 3i Marianas wide field live-cell imaging workstation (100×, 1.45 NA oil immersion objective). Sif velocity and lifespan measurements were collected from 22 individual cells by time-lapse imaging at one frame/second for 25 min. To avoid phototoxicity effects, only the first 5 min of each time series was used for velocity measurements for all Sifs. For lifespan measurements, the >600 seconds category included all tubules that formed within the first 5 min of imaging and could be clearly identified in ≥600 consecutive frames (at one frame/second). Many long-lived Sifs persisted until the end of the recording period, so the values provided for their lifespans represent a minimum estimate. Shorter lived tubules (<600 seconds) were those that appeared at any time during imaging and persisted for >25 but <600 seconds. Only tubules that could be traced back to the SCV were scored. Tubule loss was defined as complete retraction back to the parent compartment.

Time-lapse movies were assembled using ImageJ (written by Wayne Rasband at the U.S. National Institutes of Health and available by anonymous FTP from zippy.nimh.nih.gov). Sif velocities were quantified using ImageJ macro Manual Tracking. All figures were constructed using Volocity(Improvision) and AdobePhotoshop7.0 (Adobe Systems).

### Immunofluorescence microscopy

HeLa cells grown on coverslips in 24-well dishes were infected with mCherry-*Salmonella*SL1344 and fixed at indicated time-points p.i. in 2.5% paraformaldehyde in PBS at 37°C for 10 min. Cells were washed in PBS and permeabilized in PBS containing 10% normal donkey serum and 0.1% saponin for 10 min at room temperature. Cells were probed with mouse anti-β-tubulin (Sigma; clone SAP.4G5; 1:400) and rabbit anti-LAMP1 (Abcam 19294; 1:1000) and subsequently Cy5-conjugated anti-mouse secondary (Jackson ImmunoResearch Laboratories, Inc.; 1:400) and Alexa-488 anti-rabbit secondary (1:400) antibodies. For HeLa cells expressing LAMP1-mGFP, only detection with anti-β-tubulin was performed. Coverslips were mounted blind in either Mowiol or ProLong Gold Antifade (Invitrogen). Quantitative measurements were determined from images taken on the spinning disk microscope described above. Images shown are *z*-projections of scanning confocal stacks taken on a Zeiss LSM510. To quantify the effect of microtubule depolymerization on Sif stability, the percent of infected cells containing at least one LAMP1-positive tubule was enumerated for cells incubated with or without nocodazole treatment as described above. Values are the mean ± SD from three independent experiments.

### Estimation of SEI and Sif motility

Estimates of SEI in fixed cells were made as described previously (8). Briefly, cells infected with *Salmonella*were fixed and processed for microscopy using anti-β-tubulin antibodies to stain microtubules. Sifs were revealed either by staining with anti-LAMP1 antibodies or by presence of LAMP1-mGFP in transfected cells. *Salmonella*not expressing mCherry or mRFP were revealed using anti-LPS antibodies. For live cell estimates of SEI (data not shown), cell margins were detected using DIC microscopy, *Salmonella*were detected using mRFP, and Sifs were labeled with LAMP1-mGFP. Infected cells were selected based on the presence of bacteria, and then, images from a single focal plane (epifluorescence), or a *z*-series projection (confocal), were obtained for each wavelength (bacteria, LAMP1 and microtubules or DIC). Using Metamorphsoftware, the total microtubule area for each cell was determined after defining the perimeter of β-tubulin staining; total cell areas were determined from the DIC images. Subsequently, the perimeter of the outermost point of all LAMP1-decorated Sifs was drawn. For SEI estimates in fixed cells, the LAMP1 area was then divided by the β-tubulin or entire cell area and multiplied by 100 to give a percentage that represents the SEI.

Sif motility was scored in a random sampling of infected cells. A cell containing LAMP1-mGFP-labeled Sifs was viewed using fluorescence optics for 1 min. Cells exhibiting any Sif tubule extension, retraction or branching in this interval were scored positive for motility.

## Supporting Information

**Movie S1: Time-lapse series showing Sif dynamics in LAMP1-mGFP (green)-transfected HeLa cells infected with cherry-Salmonella (red).** Confocal live cell imaging started 6 h p.i. acquiring single optical planes every 0.7 seconds. Scale bar = 10 µm.

**Movie S2: Time-lapse series of Sif dynamics in the absence of micro-tubules.** HeLa cells were infected with cherry-Salmonella (red) for 6 h and then 16 mM nocodazole added for 1 h before live cell imaging. The endocytic pathway was loaded with dex-AF488 (green) before and throughout infection to visualize Sifs. Confocal live cell imaging started 7 h p.i., and single optical planes were collected every 0.7 seconds. Scale bar = 10 µm.

**Movie S3: Time-lapse series showing delivery of dex-AF488 from an endocytic vesicle to a pre-existing Sif tubule.** Confocal live cell imaging started 6.5 h p.i. acquiring single optical planes every 0.9 seconds. Scale bar = 10 µm.
